# Beyond engagement: Interactive response systems, student attitudes, and technology acceptance in University EFL classrooms

**DOI:** 10.1371/journal.pone.0360411

**Published:** 2026-10-08

**Authors:** Ge Song, Siti Hajar Halili, Zamzami Zainuddin

**Affiliations:** 1 Department of Curriculum and Instructional Technology, Faculty of Education, University of Malaya, Kuala Lumpur, Malaysia; 2 Flinders University Institute for Mental Health and Wellbeing, Flinders University, Adelaide, SA, Australia; Guangdong University of Foreign Studies, CHINA

## Abstract

Interactive Response Systems (IRS) are increasingly used in English as a Foreign Language (EFL) classrooms to support participation, feedback, and interaction. However, less is known about how IRS-supported instruction influences students’ broader classroom attitudes and how students’ acceptance of IRS can be explained from a technology-acceptance perspective. This study examined whether changes in university EFL students’ classroom attitudes differed between an IRS-supported intact class and a traditional-instruction intact class, and investigated whether perceived usefulness, perceived ease of use, and prior technological experience predicted students’ attitudes toward IRS. A quasi-experimental pre-test-post-test control-group design was adopted with 102 undergraduate students at a Chinese university. One intact class received IRS-supported instruction through the Unipus platform (n = 51), while another received traditional instruction without IRS integration (n = 51). Student attitudes were measured across eight dimensions: learning atmosphere, participation, attention, confidence, interactivity with teacher, interactivity with peers, learning support, and learning achievement/satisfaction. Mixed-design ANOVA showed significant Group × Time interaction effects across all dimensions, indicating greater attitudinal improvement in the IRS-supported group. Follow-up post-test comparisons also showed significantly more positive attitudes in the IRS-supported group, with medium to large effect sizes. Regression analysis showed that perceived usefulness and perceived ease of use significantly predicted attitudes toward IRS, whereas prior technological experience did not. The model explained 40.2% of the variance. These findings suggest that IRS-supported instruction may provide additional pedagogical support for improving EFL students’ classroom attitudes when meaningfully integrated into lesson design.

## Introduction

In recent years, educational technologies have become more common in English as a Foreign Language (EFL) classroom, enabling a shift from teacher-centred to more interactive and learner-centred approaches. The main issue is not access to technology, but how meaningfully it is integrated to support classroom learning and interaction. In many EFL contexts, particularly exam-driven, hierarchical systems, technology use remains superficial, providing little pedagogical transformation [1]. For instance, digital projectors are often limited to displaying teacher-made grammar slides; interactive whiteboards are used mainly as screens for textbook explanations; and student clickers simply record quiz answers without promoting discussion or collaboration. These practices reinforce teacher control, restrict student engagement, and prevent technology from fostering genuine interaction or learner autonomy. This challenge is especially evident in China, where traditional classroom dynamics and teacher-led instruction still dominate [2].

Interactive Response Systems (IRS), also known as clickers or instant response systems, have become key tools in language education [3]. IRS boosts participation, delivers immediate feedback, and helps create more interactive classrooms [4,5]. Research shows the IRS can improve engagement, self-efficacy, and learning [5]. These systems reduce student anxiety by making answers anonymous and participation safer [6,7]. Still, most studies focus only on whether IRS increases engagement or performance. Few examine how students experience and assess IRS in EFL classrooms. Here, ‘student experience’ includes motivation, anxiety, confidence, satisfaction, willingness to participate, and feelings of inclusion in classroom interactions. Aspects such as students’ motivation, anxiety, satisfaction, and perceptions of classroom support remain underexplored in IRS research [3]. This has left a gap in instructors’ understanding of the IRS’s broader effects on learners.

Clarifying this issue is crucial. In language classrooms, the impact of technology reaches beyond academic improvement. Emotional factors such as anxiety, confidence, and willingness to participate influence EFL learning, along with perceptions of classroom support. The IRS is thought to foster more inclusive and responsive environments, but little research examines its effects on student attitudes in areas such as classroom environment, attention, participation, confidence, interaction, and satisfaction. As a result, even though the IRS might boost engagement, we know little about its wider impact on classroom attitudes.

Another related gap is the lack of a theory-based explanation for students’ positive attitudes toward IRS. Most studies on IRS in EFL focus on results rather than the reasons for technology acceptance [1,8]. Few have considered whether perceived usefulness (PU), perceived ease of use (PEOU), and prior technology experience shape attitudes toward IRS. The Technology Acceptance Model (TAM) is useful in EFL settings. It explains how the benefits and ease of educational technology address problems like student anxiety, low participation, and limited engagement. According to TAM, technology must be both useful and easy to use in real classrooms for positive adoption [9]. In EFL classrooms, anxiety and reluctance often hinder students [10]. Perceived usefulness helps them see technology as a support for language learning and classroom interaction. Perceived ease of use can lower apprehension and help students feel confident using technology. Prior technology experience may also reduce barriers to participation [11]. Clarifying these factors is vital for understanding IRS adoption and addressing the challenges faced by EFL instructors and students.

Although previous studies have examined the use of IRS in relation to engagement, motivation, participation, anxiety reduction, and learning outcomes, several issues remain insufficiently addressed in the EFL literature. First, existing studies often focus on selected outcomes rather than treating students’ classroom attitudes toward IRS-supported instruction as a multidimensional construct. As a result, less is known about how IRS-supported instruction relates simultaneously to learning atmosphere, participation, attention, confidence, teacher-related interactivity, peer-related interactivity, learning support, and learning achievement/satisfaction. Second, much of the existing IRS research describes positive classroom effects, but provides less explanation of why students develop favourable attitudes toward IRS from a technology-acceptance perspective. In particular, the roles of perceived usefulness, perceived ease of use, and prior technological experience remain underexamined in IRS-supported EFL classrooms. Third, because EFL learning is shaped by affective and interactional factors such as participation pressure, confidence, and fear of making mistakes, technology acceptance in this context needs to be interpreted in relation to classroom interaction rather than only as a general issue of system adoption.

This study therefore examines IRS-supported instruction in university EFL classrooms from both an attitudinal and technology-acceptance perspective. It investigates whether IRS-supported instruction is associated with changes in students’ classroom attitudes across multiple dimensions and whether perceived usefulness, perceived ease of use, and prior technological experience predict students’ attitudes toward IRS. With these aims in mind, the study poses the following research questions:

1. To what extent does IRS integration improve students’ perceptions of classroom atmosphere, participation, attention, and satisfaction?
2. To what extent do perceived usefulness, perceived ease of use, and prior technological experience predict students’ attitudes toward IRS in EFL classrooms?

This study contributes to the literature in three ways. First, it conceptualises students’ attitudes toward IRS-supported EFL learning as a multidimensional classroom construct rather than as a single indicator of engagement or satisfaction. Second, it connects IRS-supported EFL instruction with the Technology Acceptance Model by examining whether perceived usefulness, perceived ease of use, and prior technological experience explain students’ attitudes toward IRS. Third, it provides quasi-experimental evidence from a university EFL context, showing how IRS-supported instruction may provide additional pedagogical support for classroom participation, interaction, feedback, and learning support when integrated into lesson design.

## Literature review

### Interactive response systems in education

IRS, also known as clickers, classroom response systems, or student response systems, have been widely adopted in higher education to support active learning and classroom interaction. Across disciplines, prior research has generally associated IRS with increased participation, attention, motivation, and engagement, especially in classes where students may otherwise remain passive or where individual contributions are difficult to elicit [12–17]. These systems allow instructors to pose questions, collect responses in real time, display class-level response patterns, and provide immediate feedback [4,12]. In this sense, IRS can make student thinking more visible and create opportunities for more responsive teaching.

At the same time, the educational value of IRS should not be understood as automatic or uniform. Previous studies suggest that IRS can support learning performance, conceptual understanding, and retention when it is used for formative assessment, peer discussion, and clarification of misconceptions [15,18]. However, evidence across fields such as engineering and mathematics has also shown mixed or context-dependent effects, partly because studies differ in learning outcomes, assessment methods, task design, and duration [19,20]. This suggests that IRS effectiveness depends not simply on the presence of a response tool, but on how the tool is integrated into teaching and how response data are used to guide learning.

Recent developments have shifted IRS use from hardware clickers to mobile and web-based platforms such as Mentimeter, Kahoot!, Poll Everywhere, and Plickers. These platforms broaden the forms of classroom response by supporting polls, quizzes, open-ended answers, word clouds, and gamified interaction [21–23]. Such tools may increase immediacy and enjoyment, but their pedagogical value depends on whether they are aligned with instructional goals. When response activities are used only for novelty or simple answer collection, they may have limited impact. By contrast, when they are connected to feedback, discussion, and reflection, they can support more meaningful classroom interaction [24–26].

A further issue concerns the social and affective role of IRS. Studies have shown that anonymous or semi-anonymous responding can reduce fear of public error and encourage participation from students who are less confident or reluctant to speak [20,27]. However, these benefits may be constrained by technical problems, unequal access to devices or internet connectivity, insufficient teacher preparation, or poorly designed questions [25,28,29]. Therefore, the general IRS literature indicates both promise and caution: IRS can support participation, feedback, and interaction, but its effects are mediated by platform choice, task design, teacher facilitation, and classroom context.

Overall, existing research provides strong evidence that IRS can enhance engagement and support active learning in higher education. Nevertheless, much of this literature focuses on general participation, attention, achievement, or performance outcomes. Less attention has been given to how students evaluate IRS-supported learning as a broader classroom experience involving atmosphere, confidence, interaction, support, and satisfaction. This limitation is particularly important for EFL classrooms, where affective and interactional conditions strongly shape students’ willingness to participate.

### IRS in English as a Foreign Language (EFL) classrooms

EFL classrooms provide a particularly relevant context for examining the pedagogical value of Interactive Response Systems because language learning is shaped not only by cognitive processing, but also by affective and interactional conditions. Many EFL learners experience communication anxiety, reluctance to speak, fear of negative evaluation, and limited confidence when participating in classroom interaction [2,10]. In such contexts, IRS may be useful because it can lower the threshold for participation by allowing students to respond quickly, anonymously or semi-anonymously, and with immediate feedback. Therefore, the potential value of IRS in EFL learning lies not only in increasing the number of responses, but also in changing how students experience participation, feedback, and classroom interaction.

Previous EFL studies have generally reported positive effects of IRS on motivation, engagement, participation, and classroom interaction [15,30]. Some studies have shown that IRS-supported activities can encourage more active involvement in grammar, vocabulary, reading, and comprehension tasks, particularly when response activities are combined with teacher feedback and peer discussion [12,31]. Other research has highlighted the affective value of IRS, suggesting that anonymous response functions can reduce anxiety and make participation feel safer for learners who may hesitate to answer publicly [6,27,30]. These findings are important because they suggest that IRS may support both behavioural participation and affective readiness to participate in language classrooms.

However, the EFL evidence also shows considerable variation across platforms, instructional designs, and measured outcomes. Studies using tools such as Poll Everywhere, Mentimeter, Pear Deck, Kahoot!, and other response systems have reported improvements in engagement, enjoyment, interaction, or perceived learning, but these effects are often tied to specific classroom tasks and implementation conditions [22,27,32–34]. Low-tech alternatives such as mini whiteboards have also been found to reproduce some participation and attitudinal benefits, suggesting that the pedagogical structure of response activities may matter as much as the technological sophistication of the tool [35]. Similarly, in online or blended EFL settings, the effectiveness of IRS may be constrained by connectivity, device access, teacher facilitation, and the quality of question design [25,36]. These findings indicate that IRS should not be treated as a universally effective tool; rather, its value depends on how response opportunities are embedded into language learning activities.

Another important strand of research connects IRS-supported learning with willingness to communicate, anxiety reduction, confidence, and broader communicative engagement. IRS-supported pair work and response-based discussion may provide students with a lower-pressure route into classroom communication, especially when learners can first submit responses digitally before engaging in oral interaction [6]. More broadly, research on technology-mediated language learning suggests that supportive, multimodal, and low-risk environments can increase willingness to communicate while reducing anxiety, boredom, or fear of participation [37–41]. Although these studies do not all focus specifically on IRS, they help explain why response systems may be pedagogically relevant in EFL classrooms: they can provide structured opportunities for participation, feedback, and confidence-building within ordinary lesson routines.

Taken together, the EFL literature suggests that IRS can support participation, motivation, interaction, and reduced anxiety. Nevertheless, the evidence remains fragmented. Some studies focus on willingness to communicate or anxiety, others on engagement, enjoyment, achievement, or perceived learning. Fewer studies examine students’ classroom attitudes toward IRS-supported instruction across multiple affective, behavioural, interactional, and pedagogical dimensions. In addition, relatively limited attention has been given to why students develop favourable attitudes toward IRS from a technology-acceptance perspective. This limitation is important because students’ positive responses to IRS may depend not only on the tool itself, but also on whether they perceive it as useful, manageable, and meaningful for EFL learning.

### IRS and the flipped classroom model

The flipped classroom provides a relevant pedagogical context for integrating IRS into EFL instruction. By relocating part of content preparation to the pre-class stage, the flipped model creates more in-class time for interaction, discussion, feedback, and collaborative learning [42,43]. Within this structure, IRS can serve as a practical tool for checking preparation, eliciting responses, identifying misunderstandings, and guiding follow-up discussion [6,21–23]. Therefore, IRS is not merely a technological add-on in flipped learning, but can function as a classroom mechanism that connects pre-class preparation with in-class participation and feedback.

Previous studies suggest that IRS-supported flipped instruction can enhance student engagement, motivation, and participation when response activities are closely aligned with learning objectives [6,42–44]. In EFL classrooms, this integration may be particularly useful because learners often need structured opportunities to practise language, receive immediate feedback, and participate in lower-pressure interaction [6,27,30]. IRS activities such as polls, comprehension checks, short quizzes, and live responses can make students’ understanding more visible and provide teachers with real-time information for adjusting instruction [15,21–24].

However, the benefits of IRS-supported flipped learning are not automatic. Prior research has shown that its effectiveness depends on task design, student preparation, teacher facilitation, technological accessibility, and the meaningful use of response data [5,25,35,45]. If IRS activities are used only for novelty or simple answer collection, they may have limited pedagogical value. For this reason, the present study treats IRS as part of instructional design rather than as a standalone technology. The focus is not on the flipped classroom model itself, but on how IRS-supported instruction may influence students’ classroom attitudes and how students’ acceptance of IRS can be explained through TAM-related factors.

### Student attitudes, technology acceptance, and the TAM framework

Students’ attitudes toward classroom technology are shaped by both their perceptions of the tool and the learning conditions in which the tool is used. In educational technology research, the TAM has been widely used to explain how learners evaluate and adopt digital tools. TAM proposes that perceived usefulness and perceived ease of use are central to users’ attitudes toward technology and their willingness to use it [9]. In higher education, these constructs have been applied to explain students’ acceptance of e-learning systems, learning management systems, mobile learning tools, MOOCs, and other digital platforms [46–52]. Across these contexts, students are more likely to respond positively when they perceive a technology as helpful for learning and manageable to use.

However, applying TAM to IRS-supported EFL classrooms requires attention to the specific pedagogical and interactional context of language learning. IRS differs from many educational technologies because it is embedded directly into real-time classroom interaction. Students do not evaluate IRS only as a technical system; they also evaluate whether it helps them participate, receive feedback, check understanding, and interact with teachers and peers [5–7,28,30,33]. In this sense, perceived usefulness in an EFL classroom may involve more than efficiency or performance improvement. It may also involve whether the tool reduces participation pressure, supports comprehension, and makes classroom communication more accessible.

Perceived ease of use also has a specific meaning in EFL learning. If a response system is difficult to operate or interrupts the flow of classroom tasks, it may distract learners from language use and reduce its pedagogical value. By contrast, when the system is easy to access and simple to use, students can focus more on responding to questions, comparing answers, and engaging with feedback. Previous studies on IRS and related classroom response tools have similarly suggested that students’ positive evaluations depend not only on the availability of technology, but also on whether the tool is convenient, accessible, and pedagogically meaningful in classroom practice [28,33,34,46–52].

Prior technological experience may also influence students’ initial readiness to use classroom technologies. Learners who are more familiar with digital tools may feel less anxious when using a response system. However, previous technology experience alone may not be sufficient to explain students’ attitudes toward IRS. In classroom-based EFL learning, students’ evaluations are likely to depend more directly on whether the system is experienced as useful, easy to use, and supportive of actual learning activities [53–55]. This means that technology acceptance should be interpreted in relation to situated classroom experience rather than general digital familiarity alone.

This suggests that TAM provides a useful but incomplete lens for understanding IRS-supported EFL learning. While perceived usefulness and perceived ease of use help explain students’ acceptance of classroom technology, EFL learners’ attitudes are also shaped by affective and interactional conditions, such as confidence, participation pressure, fear of negative evaluation, feedback opportunities, and teacher-student or peer interaction [37–41,56]. Therefore, the present study applies TAM not as a purely technical adoption model, but as a framework for examining how students evaluate IRS within the specific social and pedagogical conditions of university EFL classrooms.

### Synthesis of the literature and research gap

Overall, the literature suggests that IRS can support participation, feedback, engagement, and interaction in higher education and EFL classrooms. However, these benefits are not automatic. Across previous studies, the effectiveness of IRS appears to depend on instructional design, platform accessibility, question quality, feedback use, teacher facilitation, and students’ classroom context [5,21–29,35]. This indicates that IRS should not be understood simply as a technology that increases participation, but as a pedagogical tool whose value depends on how it is integrated into classroom learning.

In EFL contexts, prior research has shown that response systems may help reduce participation pressure, support interaction, and increase students’ willingness to engage in classroom activities [6,27,30,37–41]. Nevertheless, the existing evidence remains fragmented. Some studies focus on motivation, anxiety, willingness to communicate, or achievement, while others examine engagement, enjoyment, perceived learning, or technology acceptance. Fewer studies examine students’ attitudes toward IRS-supported EFL instruction as a multidimensional classroom construct that includes learning atmosphere, participation, attention, confidence, teacher-related interactivity, peer-related interactivity, learning support, and learning achievement/satisfaction.

A second gap concerns the explanation of students’ attitudes toward IRS. Although TAM has been widely used to explain technology acceptance in higher education, fewer studies have applied TAM to explain students’ attitudes toward IRS within classroom-based EFL learning [9,46–56]. This distinction matters because students’ acceptance of IRS in EFL classrooms may be shaped not only by perceived usefulness and ease of use, but also by affective and interactional conditions such as confidence, participation pressure, feedback opportunities, and classroom communication.

The present study addresses these gaps by examining IRS-supported instruction from both a multidimensional attitude perspective and a technology-acceptance perspective. Specifically, it investigates whether IRS-supported instruction is associated with changes in university EFL students’ classroom attitudes and whether perceived usefulness, perceived ease of use, and prior technological experience predict students’ attitudes toward IRS.

## Methodology

### Research design

This study employed a quasi-experimental pre-test-post-test control-group design to investigate university EFL students’ attitudes toward IRS. Two intact EFL classes were used: one class served as the IRS-supported condition, while the other served as the traditional instruction condition. Individual random assignment was not implemented because the study was conducted in naturally existing university classes for instructional and administrative reasons. Therefore, the design is more accurately described as quasi-experimental rather than fully experimental. Importantly, because each instructional condition was represented by only one intact class, class membership was fully confounded with instructional condition.

The pre-test-post-test structure enabled the study to compare patterns of attitudinal change over time between the two intact classes. Both classes completed the same attitude questionnaire before and after the instructional period. The IRS-supported class received instruction that integrated the Unipus-based IRS into classroom activities, whereas the traditional instruction class received the same course content without IRS integration. Both classes were taught by the same instructor to reduce potential variation associated with teaching style, classroom management, and instructional delivery. However, because only one class represented each condition, unmeasured class-specific influences could not be statistically separated from the instructional condition.

Accordingly, the design allowed the study to examine whether the pattern of attitudinal change differed between the two intact classes under the two instructional conditions. The findings are therefore interpreted as evidence of differential change between these classes rather than as an estimate of a class-independent causal treatment effect.

### Participants

The participants were 102 undergraduate students enrolled in compulsory EFL courses at a comprehensive university in China. All participants were non-English majors, reflecting the typical composition of university EFL classrooms in this context. Two intact classes participated in the study. One class served as the IRS-supported group (n = 51), while the other class served as the traditional instruction group (n = 51).

As shown in Table 1, the two groups were comparable in age, gender distribution, and baseline English performance. Students’ previous-semester English course scores were used as an indicator of baseline English proficiency. The difference between the IRS-supported group and the traditional instruction group in previous English scores was not statistically significant, t(100) = 1.57, p = .121, suggesting that the two groups were broadly comparable before the intervention.

**Table 1:** Demographic and baseline characteristics of participants.

Characteristic	IRS-supported group (n = 51)	Traditional instruction group (n = 51)	Total (N = 102)	Group comparison
**Age, years, M (SD)**	20.02 (0.62)	20.02 (0.47)	20.02 (0.54)	t(100) = 0.00, p = 1.000
**Age range, years**	19–22	19–21	19–22	—
**Gender, n (%)**				χ²(1) = 0.16, p = .692
**Female**	28 (54.9)	26 (51.0)	54 (52.9)	—
**Male**	23 (45.1)	25 (49.0)	48 (47.1)	—
**Previous English score, M (SD)**	83.49 (4.84)	81.94 (5.15)	82.72 (5.03)	t(100) = 1.57, p = .121
**Previous English score range**	71–93	70–92	70–93	—

The study used intact class sampling rather than individual random assignment because the intervention was implemented within naturally existing university classes, and reassigning students into new groups was not feasible for instructional and administrative reasons. Therefore, the sample and grouping procedure were consistent with the quasi-experimental nature of the study. To reduce potential instructional variation, both classes were taught by the same instructor, followed the same compulsory EFL syllabus, and used the same course materials. The key difference between the two groups was that the IRS-supported group used the Unipus-based Interactive Response System during classroom activities, whereas the traditional instruction group received the same learning content without IRS integration.

### Ethical considerations

Ethical approval for the study was granted by the University of Malaya Research Ethics Committee (UMREC; Approval No. UM.TNC(P&I)/UMREC_5128). Local institutional permission to conduct the study was also obtained from the participating university in China before data collection. The study was conducted in accordance with the approved ethical procedures for research involving human participants. Before data collection, students were informed of the purpose of the study, the voluntary nature of participation, the anonymity of questionnaire responses, and their right to withdraw from the study at any time without penalty.

Verbal informed consent was obtained from all participants before they completed the questionnaires. The use of verbal consent was approved as part of the ethical procedure because the questionnaire data were collected anonymously and no personally identifiable information was included in the analytical dataset. The consent procedure was conducted before the pre-test questionnaire was administered. Students were provided with an explanation of the study and were asked to indicate verbally whether they agreed to participate. Only students who provided verbal consent completed the questionnaires.

The consent process was documented by the researcher through a data collection record indicating that the consent procedure had been completed before questionnaire administration. The process was witnessed during the classroom data collection session. No names or personally identifiable signatures were collected in order to preserve participant anonymity. All questionnaire responses were coded using anonymous student IDs and were used only for research purposes.

### Instruments

#### Student attitude scale.

Students’ classroom attitudes were measured using a 40-item Student Attitude Scale constructed for the present study. The eight conceptual dimensions and the item content were informed by recurring themes in previous research on classroom and audience response systems, including classroom atmosphere, participation, attention, confidence, interaction, learning support, and student satisfaction [57,58]. The scale was not adopted from a single existing 40-item instrument. All 40 items were developed specifically for the present study rather than reproduced verbatim from the cited sources. Instead, the literature informed the conceptual structure and the development of context-specific items for university EFL learning and IRS-supported classroom activities.

The final scale consisted of eight dimensions: learning atmosphere, participation, attention, confidence, teacher-student interaction, peer interaction, learning support, and learning achievement/satisfaction. Each dimension contained five items, resulting in 40 items in total. All items were rated on a five-point Likert scale ranging from 1 (strongly disagree) to 5 (strongly agree). Higher scores indicated more positive attitudes toward classroom learning.

The questionnaire was administered directly in English. Because the participants were university EFL students, the items were written in clear and accessible English to minimise unnecessary linguistic difficulty while preserving the intended construct meaning. No translation or back-translation procedure was used because the English-language questionnaire was administered directly. Table 2 presents the structure of the scale and one representative item from each dimension.

**Table 2:** Structure of the student attitude scale.

Dimension	Number of items	Sample item
**Learning atmosphere**	5	The classroom atmosphere was active and supportive during English learning.
**Participation**	5	I was willing to participate in English classroom activities.
**Attention**	5	The classroom activities helped me pay attention during English lessons.
**Confidence**	5	I felt more confident when taking part in English classroom learning.
**Interactivity (Teacher)**	5	The classroom activities helped me interact with the teacher.
**Interactivity (Peers)**	5	The classroom activities encouraged me to interact with my classmates.
**Learning support**	5	The classroom activities provided useful support for my English learning.
**Learning achievement/satis.**	5	I was satisfied with what I learned in the English class.

#### Technology acceptance measures.

To examine the predictors of students’ attitudes toward the IRS, a set of TAM-related measures was administered to students in the IRS-supported group after the intervention. The measures were developed and adapted based on the core TAM constructs of perceived usefulness and perceived ease of use [9], as well as prior educational technology acceptance research [46–52]. Because students in the traditional instruction group did not use the IRS during the intervention, they did not complete these IRS-specific measures. Therefore, the regression analysis was conducted only with the IRS-supported group.

The TAM-related measures included four constructs: perceived usefulness, perceived ease of use, prior technological experience, and overall attitude toward IRS. As shown in Table 3, each construct contained three items and was rated on a five-point Likert scale ranging from 1 (strongly disagree) to 5 (strongly agree). Perceived usefulness referred to the extent to which students believed that the IRS supported their English learning, classroom participation, and understanding of lesson content. Perceived ease of use referred to the extent to which students perceived the IRS as clear, manageable, and easy to operate during classroom activities. Prior technological experience was included as an additional background predictor to assess students’ general familiarity with educational technologies before using the IRS. Overall attitude toward IRS measured students’ general evaluative response to using the IRS in the EFL classroom.

**Table 3:** Technology acceptance measures.

Construct	Number of items	Measurement focus	Cronbach’s alpha
**Perceived usefulness**	3	Perceived value of IRS for supporting English learning, classroom participation, and understanding of lesson content	.86
**Perceived ease of use**	3	Perceived clarity, convenience, and ease of operating IRS during classroom activities	.88
**Prior technological experience**	3	Students’ general familiarity with educational technologies before using IRS	.77
**Overall attitude toward IRS**	3	Students’ general evaluative attitude toward IRS-supported EFL learning	.81

The wording of the items was adapted to the university EFL context and the classroom use of the Unipus-based IRS. Perceived usefulness items focused on whether the IRS helped students participate, understand lesson content, and receive learning support. Perceived ease of use items focused on whether the IRS was easy to operate, clear to follow, and convenient to use during class. Prior technological experience items focused on students’ previous familiarity with educational technologies, while attitude items measured their overall positive evaluation of IRS-supported learning.

The internal consistency of the four measures was acceptable to high. Cronbach’s alpha was .86 for perceived usefulness, .88 for perceived ease of use, .77 for prior technological experience, and .81 for overall attitude toward IRS. These reliability values indicated that the measures were suitable for use in the subsequent regression analysis.

#### Validity and reliability.

Because the Student Attitude Scale was constructed for the present study, its measurement evidence was treated as preliminary. The items were organised according to eight a priori conceptual dimensions and were reviewed for clarity, conceptual relevance, redundancy, and contextual appropriateness for university EFL learning and IRS-supported classroom interaction.

The dimensional structure was explored using students’ post-test responses to the 40 items. Principal component extraction with orthogonal Varimax rotation was used. The Kaiser-Meyer-Olkin (KMO) measure of sampling adequacy was .62, and Bartlett’s test of sphericity was significant, χ²(780) = 1804.22, p < .001, indicating sufficient inter-item correlations for exploratory analysis. An eight-component solution was retained based on the prespecified eight-dimensional conceptual structure, inspection of the scree plot, which showed a marked decline after the eighth component, and the interpretability of the rotated solution, in which the five items designed for each dimension loaded most strongly on the same component. The eight rotated components jointly explained 60.24% of the total variance. The five items designed for each dimension had their largest loading on the same rotated component, and the primary rotated loadings ranged from .63 to .81.

Reliability was assessed using Cronbach’s alpha. As shown in Table 4, the coefficients for the eight dimensions ranged from .77 to .85, indicating acceptable to good internal consistency. Given the modest sample size (N = 102) relative to the 40-item scale and the KMO value of .62, the factor-analytic results should be interpreted cautiously as preliminary rather than confirmatory evidence for the proposed eight-dimensional structure. Replication with larger independent samples is needed to establish the stability of this structure.

**Table 4:** Reliability and exploratory factor analysis results for the student attitude scale.

Dimension	Number of items	Cronbach’s alpha	Rotated factor loading range
**Learning atmosphere**	5	.80	.68–.75
**Participation**	5	.85	.70–.80
**Attention**	5	.77	.66–.75
**Confidence**	5	.79	.66–.79
**Interactivity (Teacher)**	5	.82	.66–.78
**Interactivity (Peers)**	5	.81	.64–.81
**Learning support**	5	.81	.63–.81
**Learning achievement/satis.**	5	.80	.66–.77

### Procedure

Data were collected between October 13, 2025 and November 14, 2025. At the beginning of the study, both the IRS-supported group and the traditional instruction group completed the pre-test questionnaire to measure their baseline classroom attitudes. As shown in Table 5, the intervention was then implemented over a four-week instructional period. At the end of the intervention, both groups completed the same questionnaire as the post-test.

**Table 5:** Implementation of IRS-supported and traditional instruction.

Component	IRS-supported group	Traditional instruction group
**Duration**	Four weeks	Four weeks
**Course content**	Same compulsory EFL course content	Same compulsory EFL course content
**Instructor**	Same instructor	Same instructor
**Platform**	Unipus-based Interactive Response System	No IRS platform
**Main classroom format**	IRS-supported quizzes, polls, comprehension checks, live responses, and guided discussion	Teacher-led questioning, oral responses, and conventional classroom discussion
**Question formats**	Multiple-choice questions, short-answer responses, polls, and live response activities	Oral questions and conventional discussion prompts
**Feedback mode**	Immediate class-level feedback based on IRS response patterns	Teacher-led feedback through explanation and correction
**Peer discussion**	Triggered by IRS response patterns, especially when disagreement or misunderstanding appeared	Conducted through conventional classroom interaction
**Instructional purpose**	To support participation, immediate feedback, comprehension checking, and classroom interaction	To cover the same learning content without IRS-supported interaction

Both groups followed the same compulsory EFL course syllabus and were taught by the same instructor. The same course topics, textbook-based learning content, and classroom learning objectives were used for both groups. The main difference between the two groups was the mode of classroom interaction. The IRS-supported group used the Unipus-based Interactive Response System during classroom activities, whereas the traditional instruction group received the same instructional content through teacher-led questioning, oral discussion, and conventional classroom interaction without IRS integration.

In the IRS-supported group, IRS activities were embedded into different phases of each lesson. At the beginning of the lesson, short polls or warm-up questions were used to activate students’ prior knowledge and introduce the lesson topic. During the lesson, comprehension-check questions and short quizzes were used to monitor students’ understanding of vocabulary, grammar, reading content, or topic-related ideas. After students submitted responses through the Unipus platform, the instructor displayed or reviewed class-level response patterns and used them to provide immediate feedback, clarify misunderstandings, and guide follow-up explanation or discussion. Toward the end of the lesson, brief review questions or summary checks were used to consolidate learning and encourage students to reflect on key points.

The IRS question formats included multiple-choice questions, short-answer responses, polls, and live response activities. These formats were selected because they allowed students to participate quickly and, where appropriate, with reduced pressure compared with public oral responses. Peer discussion was also encouraged when response patterns showed disagreement or misunderstanding. In such cases, students were invited to compare answers, explain their reasoning, and reconsider their responses before the teacher provided clarification.

The traditional instruction group covered the same learning content but did not use the IRS platform. Students participated through conventional teacher-led questioning, whole-class discussion, and oral responses. Feedback was provided by the instructor through explanation, correction, and classroom discussion rather than through real-time digital response summaries.

### Data analysis

The data were analysed using SPSS version 26.0. Descriptive statistics, including means and standard deviations, were first calculated to summarise students’ responses across the eight attitudinal dimensions. Baseline patterns were examined by comparing the pre-test descriptive statistics of the IRS-supported and traditional instruction groups.

To provide a more rigorous test of whether change over time differed between the two intact classes in the pre-test-post-test control-group design, mixed-design analyses of variance (mixed-design ANOVAs) were conducted for each attitudinal dimension. Time, with two levels (pre-test and post-test), was treated as the within-subject factor, while group, with two levels (IRS-supported instruction and traditional instruction), was treated as the between-subject factor. The Group × Time interaction was used as the primary statistical test of whether changes from pre-test to post-test differed between the two instructional conditions. Partial eta squared was reported as the effect size for the ANOVA results. Because the within-subject factor included only two time points, the assumption of sphericity was not applicable.

After the mixed-design ANOVAs, follow-up independent-samples t-tests were conducted to compare post-test scores between the two classes, and paired-samples t-tests were conducted to describe pre-test-post-test changes within each class. These analyses were treated as supplementary follow-up analyses, while the Group × Time interaction remained the primary test of differential change between the two instructional conditions.

To control the familywise Type I error rate across the follow-up comparisons, a Bonferroni correction was applied. The complete set comprised 24 follow-up t-tests: eight post-test comparisons between the two classes, eight pre-test-post-test comparisons within the IRS-supported class, and eight pre-test-post-test comparisons within the traditional instruction class. The adjusted significance criterion was therefore α = .05/24 = .002083 (approximately .0021). All follow-up comparisons reported as statistically significant remained significant under this adjusted criterion.

To address the second research question, multiple regression analysis was conducted using data from the IRS-supported group only (n = 51). This was because students in the traditional instruction group did not use the IRS and therefore could not meaningfully evaluate the system’s perceived usefulness or perceived ease of use. In line with the Technology Acceptance Model, perceived usefulness, perceived ease of use, and prior technological experience were entered as independent variables, while students’ overall attitudes toward IRS served as the dependent variable.

Regression diagnostics were examined to assess the robustness of the model. Multicollinearity was evaluated using tolerance and variance inflation factor (VIF) values. Residual patterns were inspected to assess normality, linearity, and homoscedasticity. Influential cases were also checked to ensure that no single case substantially distorted the regression estimates.

## Results

This section presents the quantitative findings in five parts. First, descriptive statistics are reported to summarise students’ attitudes across the eight dimensions before and after the intervention. Second, mixed-design ANOVA results are presented to examine whether changes from pre-test to post-test differed between the IRS-supported group and the traditional instruction group. Third, follow-up post-test comparisons are reported to clarify between-group differences after the intervention. Fourth, follow-up within-group comparisons are briefly described to show the direction of change in each group. Finally, regression analysis and diagnostic statistics are presented to examine whether perceived usefulness, perceived ease of use, and prior technological experience predicted students’ attitudes toward IRS.

### Descriptive statistics and baseline patterns

Descriptive statistics were first calculated to summarise students’ attitudes across the eight measured dimensions before and after the intervention. As shown in Table 6, both the IRS-supported group and the traditional instruction group reported increases from pre-test to post-test across all dimensions. However, the post-test scores of the IRS-supported group were consistently higher than those of the traditional instruction group.

**Table 6:** Descriptive statistics of student attitudes by group and time.

Dimension	Exp. Pre-test M (SD)	Exp. Post-test M (SD)	Ctrl. Pre-test M (SD)	Ctrl. Post-test M (SD)
**Learning atmosphere**	2.55 (0.91)	3.46 (0.83)	2.45 (0.82)	2.70 (0.81)
**Participation**	2.61 (0.94)	3.56 (0.84)	2.35 (0.87)	2.62 (0.82)
**Attention**	2.34 (0.82)	3.26 (0.77)	2.47 (0.92)	2.73 (0.92)
**Confidence**	2.27 (0.76)	3.24 (0.76)	2.44 (0.88)	2.69 (0.87)
**Interactivity (Teacher)**	2.41 (0.90)	3.39 (0.79)	2.22 (0.81)	2.53 (0.84)
**Interactivity (Peers)**	2.49 (0.92)	3.45 (0.84)	2.36 (0.80)	2.66 (0.84)
**Learning support**	2.46 (0.82)	3.42 (0.77)	2.47 (0.97)	2.76 (0.99)
**Learning achievement/satis.**	2.38 (0.81)	3.35 (0.74)	2.48 (0.97)	2.71 (0.97)

At the pre-test stage, the two groups showed broadly comparable levels of classroom attitudes. The experimental group’s pre-test means ranged from 2.27 to 2.61, while the control group’s pre-test means ranged from 2.22 to 2.48. These scores suggest that students in both groups began the study with relatively moderate attitudes toward classroom learning, participation, interaction, and support. Although small numerical differences were observed across individual dimensions, the overall pattern indicated that the two groups started from a generally similar baseline.

At the post-test stage, a clearer difference emerged between the two groups. The IRS-supported group reported post-test means ranging from 3.24 to 3.56, whereas the traditional instruction group reported post-test means ranging from 2.53 to 2.76. Within the IRS-supported group, the highest post-test mean was found for participation (M = 3.56, SD = 0.84), followed by learning atmosphere (M = 3.46, SD = 0.83), peer interaction (M = 3.45, SD = 0.84), and learning support (M = 3.42, SD = 0.77). This pattern suggests that students who experienced IRS-supported instruction reported more favourable classroom attitudes after the intervention, particularly in relation to participation, interaction, and perceived classroom support.

The traditional instruction group also showed some improvement over time, but its post-test means remained lower than those of the IRS-supported group across all eight dimensions. This corrected descriptive pattern is important because it indicates that the control group did not remain completely unchanged. Rather, both groups showed positive movement from pre-test to post-test, while the IRS-supported group demonstrated a more pronounced improvement.

Overall, the descriptive findings suggest that IRS-supported instruction was associated with more favourable post-test attitudes across affective, interactional, and pedagogical dimensions of classroom learning. Nevertheless, descriptive statistics alone cannot determine whether the changes over time differed significantly between the two groups. Therefore, mixed-design ANOVA was conducted in the next stage of analysis to examine the Group × Time interaction and provide a direct test of whether change over time differed between the two classes.

### Mixed-design ANOVA: Group × time effects

To examine whether changes in student attitudes differed significantly between the IRS-supported group and the traditional instruction group, mixed-design ANOVAs were conducted for each of the eight attitudinal dimensions. Time, with two levels (pre-test and post-test), was treated as the within-subject factor, while group, with two levels (IRS-supported instruction and traditional instruction), was treated as the between-subject factor. The Group × Time interaction was used as the primary indicator of whether the pattern of change over time differed between the two instructional conditions.

As shown in Table 7, significant Group × Time interaction effects were found across all eight attitudinal dimensions. The interaction was significant for learning atmosphere, F(1, 100) = 228.42, p < .001, partial η² = .696; participation, F(1, 100) = 204.71, p < .001, partial η² = .672; attention, F(1, 100) = 178.46, p < .001, partial η² = .641; confidence, F(1, 100) = 248.36, p < .001, partial η² = .713; teacher–student interaction, F(1, 100) = 204.48, p < .001, partial η² = .672; peer interaction, F(1, 100) = 211.34, p < .001, partial η² = .679; learning support, F(1, 100) = 229.19, p < .001, partial η² = .696; and learning achievement/satisfaction, F(1, 100) = 242.35, p < .001, partial η² = .708.

**Table 7:** Mixed-design ANOVA results for group × time interaction.

Dimension	df	F	p	partial η²
**Learning atmosphere**	1, 100	228.42	< .001	.696
**Participation**	1, 100	204.71	< .001	.672
**Attention**	1, 100	178.46	< .001	.641
**Confidence**	1, 100	248.36	< .001	.713
**Interactivity (Teacher)**	1, 100	204.48	< .001	.672
**Interactivity (Peers)**	1, 100	211.34	< .001	.679
**Learning support**	1, 100	229.19	< .001	.696
**Learning achievement/satis.**	1, 100	242.35	< .001	.708

The significant interaction effects indicate that the IRS-supported group demonstrated significantly greater improvement over time than the traditional instruction group across all measured dimensions. This finding provides a direct test of whether pre-test-to-post-test change differed between the two classes, rather than relying on separate within-group and between-group t-tests.

Although students in both groups reported more positive attitudes over time, the consistently significant Group × Time interactions show that the magnitude of improvement was substantially stronger in the IRS-supported group. Therefore, the results suggest that IRS-supported instruction was associated with greater positive changes in students’ classroom attitudes, particularly in relation to participation, interaction, confidence, learning support, and overall satisfaction.

### Follow-up post-test comparisons

After the mixed-design ANOVA confirmed significant Group × Time interaction effects, follow-up independent-samples t-tests were conducted to examine post-test differences between the IRS-supported group and the traditional instruction group. These analyses were used to clarify the direction and magnitude of the between-group differences at the end of the intervention.

As shown in Table 8, the IRS-supported group reported significantly higher post-test scores than the traditional instruction group across all eight attitudinal dimensions. The largest between-group difference was found for participation, t(100) = 5.72, p < .001, Cohen’s d = 1.13, indicating a large effect. Large effects were also observed for teacher–student interaction, t(100) = 5.32, p < .001, Cohen’s d = 1.05; learning atmosphere, t(100) = 4.69, p < .001, Cohen’s d = 0.93; and peer interaction, t(100) = 4.71, p < .001, Cohen’s d = 0.93.

**Table 8:** Follow-up independent-samples t-test results at post-test.

Dimension	Exp. M (SD)	Ctrl. M (SD)	t	p	Cohen’s d
**Learning atmosphere**	3.46 (0.83)	2.70 (0.81)	4.69	< .001	0.93
**Participation**	3.56 (0.84)	2.62 (0.82)	5.72	< .001	1.13
**Attention**	3.26 (0.77)	2.73 (0.92)	3.17	.002	0.63
**Confidence**	3.24 (0.76)	2.69 (0.87)	3.38	.001	0.67
**Interactivity (Teacher)**	3.39 (0.79)	2.53 (0.84)	5.32	< .001	1.05
**Interactivity (Peers)**	3.45 (0.84)	2.66 (0.84)	4.71	< .001	0.93
**Learning support**	3.42 (0.77)	2.76 (0.99)	3.76	< .001	0.75
**Learning achievement/satis.**	3.35 (0.74)	2.71 (0.97)	3.75	< .001	0.74

Moderate effects were found for learning support, t(100) = 3.76, p < .001, Cohen’s d = 0.75; learning achievement/satisfaction, t(100) = 3.75, p < .001, Cohen’s d = 0.74; confidence, t(100) = 3.38, p = .001, Cohen’s d = 0.67; and attention, t(100) = 3.17, p = .002, Cohen’s d = 0.63. These findings indicate that the IRS-supported group held more favourable post-test attitudes than the traditional instruction group, particularly in dimensions related to classroom participation and interaction.

Taken together, the follow-up post-test comparisons support the mixed-design ANOVA results by showing that students in the IRS-supported group reported significantly more positive attitudes than those in the traditional instruction group after the intervention. However, these comparisons should be interpreted as follow-up evidence, while the Group × Time interaction remains the primary test of differential change between the two classes. These follow-up comparisons were evaluated using the Bonferroni-adjusted significance criterion of α = .002083 across the complete set of 24 follow-up t-tests, and all eight post-test between-class comparisons remained statistically significant under this criterion.

### Follow-up within-group changes

Follow-up paired-samples t-tests were conducted to further describe the direction of change within each group from pre-test to post-test. These analyses were treated as supplementary follow-up evidence rather than the primary test of differential change between the two classes, because the mixed-design ANOVA provided the main test of whether changes over time differed between the two instructional conditions.

The paired-samples results showed that the IRS-supported group improved significantly from pre-test to post-test across all eight attitudinal dimensions, with t values ranging from 25.59 to 32.80, all p values < .001. The traditional instruction group also showed statistically significant pre-test to post-test improvements across the eight dimensions, with t values ranging from 6.42 to 9.95, all p values < .001. All 16 within-class comparisons also remained statistically significant under the Bonferroni-adjusted criterion of α = .002083. However, the magnitude of improvement was consistently larger in the IRS-supported group, as already reflected in the descriptive statistics and confirmed by the significant Group × Time interaction effects.

These findings clarify that the control group did not remain unchanged during the instructional period. Rather, both groups showed positive changes over time, but the IRS-supported group demonstrated substantially stronger improvement across the measured dimensions. Therefore, the within-group comparisons support, rather than replace, the mixed-design ANOVA results.

### Regression analysis and diagnostics

A multiple regression analysis was conducted to examine whether perceived usefulness, perceived ease of use, and prior technological experience predicted students’ overall attitudes toward IRS. This analysis was conducted only with the IRS-supported group (n = 51), because students in the traditional instruction group did not use the IRS and therefore could not meaningfully evaluate the system’s perceived usefulness or perceived ease of use.

The overall regression model was statistically significant, F(3, 47) = 10.54, p < .001, and explained 40.2% of the variance in students’ attitudes toward IRS (R^2^ = .402, adjusted R^2^ = .364). As shown in Table 9, perceived usefulness was the strongest predictor of students’ attitudes toward IRS (β = .43, t = 3.67, p < .001), followed by perceived ease of use (β = .35, t = 3.09, p = .003). Prior technological experience was not a statistically significant predictor (β = .15, t = 1.26, p = .213).

**Table 9:** Regression analysis and diagnostic statistics predicting student attitudes toward IRS.

Predictor	β	t	p	Tolerance	VIF
**Perceived usefulness (PU)**	.43	3.67	< .001	.93	1.08
**Perceived ease of use (PEOU)**	.35	3.09	.003	.98	1.02
**Prior technological experience**	.15	1.26	.213	.91	1.10

Note. Dependent variable = overall attitude toward IRS. Regression analysis was conducted with the IRS-supported group only (n = 51).

Regression diagnostics indicated that the assumptions of the model were acceptable. Multicollinearity was not a concern, as all VIF values were close to 1 and well below commonly used threshold values. Residual inspection did not indicate serious violations of normality, linearity, or homoscedasticity. No influential cases were identified that would substantially distort the regression estimates.

These findings indicate that students’ attitudes toward IRS were shaped primarily by their perceptions of the system’s usefulness and ease of use rather than by their general prior technological experience. In other words, students responded more positively to IRS when they perceived it as pedagogically useful and manageable during classroom learning.

## Discussion

The findings indicate that IRS-supported instruction was associated with more positive changes in students’ classroom attitudes than traditional instruction. The mixed-design ANOVA results showed significant Group × Time interaction effects across all eight attitudinal dimensions, suggesting that students in the IRS-supported group demonstrated greater improvement from pre-test to post-test than those in the traditional instruction group. This pattern provides stronger evidence than separate t-tests alone because it directly shows that the two groups differed in the magnitude of attitudinal change over time.

Importantly, the corrected results show that both groups reported more positive attitudes after the instructional period. Therefore, the findings should not be interpreted as showing that traditional instruction produced no improvement. Rather, the key result is that the IRS-supported group showed consistently larger gains across all measured dimensions. This suggests that IRS integration may have provided additional classroom support beyond the general effects of continued EFL instruction, course progression, and teacher-led classroom activities.

The strongest post-test differences were found in participation, teacher-related interactivity, learning atmosphere, and peer-related interactivity. These dimensions are closely connected to the social and interactional nature of EFL learning. In language classrooms, students’ willingness to participate can be shaped by anxiety, fear of making mistakes, limited confidence, and the perceived risk of public response. The use of IRS may have helped reduce some of these participation barriers by allowing students to respond more quickly, receive immediate feedback, and engage with classroom questions before or alongside public oral discussion. This interpretation is consistent with previous research suggesting that response systems can support participation, feedback, and more inclusive classroom interaction [6,15,27,30,33].

The findings also suggest that IRS-supported instruction may contribute to a more supportive classroom experience when it is pedagogically integrated into lesson activities. In this study, IRS activities were not used only as isolated quizzes. They were embedded into warm-up questions, comprehension checks, short quizzes, live responses, feedback, and follow-up discussion. This may help explain why students in the IRS-supported group reported stronger post-test attitudes not only in participation, but also in learning atmosphere, peer interaction, teacher interaction, learning support, and satisfaction. The results therefore point to the importance of instructional design: the value of IRS appears to depend not simply on the presence of technology, but on how it is used to organise participation, feedback, and classroom interaction.

At the same time, the findings should be interpreted cautiously. The present study used a quasi-experimental design in which one intact class represented each instructional condition. Consequently, class membership and instructional condition were fully confounded, and class-specific influences cannot be statistically separated from the observed Group × Time differences. Although the classes were taught by the same instructor and were broadly comparable on measured baseline characteristics, these features do not eliminate possible unmeasured class-level differences. The results should therefore be interpreted as evidence of differential change between the two intact classes studied rather than as proof that IRS alone caused the observed changes. Other factors, including course progression, novelty effects, and increased attention associated with participation in an intervention study, may also have contributed.

The regression findings further support the relevance of the TAM for understanding students’ attitudes toward IRS-supported EFL learning. Perceived usefulness and perceived ease of use were both significant predictors of students’ overall attitudes toward IRS, whereas prior technological experience was not significant. This suggests that students’ positive attitudes toward IRS were shaped more by their situated classroom experience with the tool than by their general familiarity with educational technology.

The significant role of perceived usefulness indicates that students responded more positively to IRS when they believed that it supported their English learning, classroom participation, understanding of lesson content, and access to feedback. In this sense, usefulness in the EFL classroom should not be interpreted only as technical efficiency. Rather, it appears to reflect students’ perception that the system helped make classroom participation and learning support more accessible. This interpretation is consistent with TAM-based research showing that perceived usefulness is central to students’ acceptance of educational technologies [9,46–52].

Perceived ease of use was also a significant predictor of students’ attitudes toward IRS. This finding suggests that students were more likely to evaluate IRS positively when they found it clear, convenient, and manageable during classroom activities. In EFL learning, ease of use is particularly important because excessive operational difficulty may distract students from language learning tasks. When the system is easy to follow, students can focus more on responding to questions, checking understanding, and participating in classroom interaction. Therefore, the present findings support the view that usability is an important condition for effective technology-supported language learning.

The non-significant effect of prior technological experience is also noteworthy. This result suggests that students’ general familiarity with educational technology did not independently predict their attitudes toward IRS once perceived usefulness and perceived ease of use were considered. In practical terms, this means that positive attitudes toward IRS may depend less on students’ prior digital background and more on whether the tool is experienced as useful and manageable in the specific classroom context. This finding reinforces the importance of pedagogical integration: even students with different levels of previous technological experience may respond positively when IRS activities are clearly connected to learning, participation, and feedback.

Overall, the TAM-related findings should be interpreted as contextual support for the applicability of TAM in IRS-supported EFL classrooms rather than as a broad theoretical extension of TAM. The study confirms that perceived usefulness and perceived ease of use remain important predictors of technology-related attitudes, while also showing that these constructs need to be understood in relation to the affective and interactional demands of language learning.

### Theoretical implications

The present study offers several theoretical implications for research on educational technology, EFL classroom interaction, and technology acceptance. First, the findings support a multidimensional understanding of students’ attitudes toward IRS-supported instruction. Previous research has often examined IRS in relation to participation, engagement, motivation, or learning performance. The present study adds to this body of work by showing that students’ responses to IRS-supported instruction can be understood across multiple classroom-related dimensions, including learning atmosphere, participation, attention, confidence, teacher-related interactivity, peer-related interactivity, learning support, and learning achievement/satisfaction. This suggests that the value of IRS in EFL learning should not be evaluated only through behavioural engagement or achievement outcomes, but also through affective, interactional, and pedagogical dimensions of classroom experience [6,15,27,30,33].

Second, the findings provide contextual support for the applicability of the Technology Acceptance Model in IRS-supported EFL classrooms. Perceived usefulness and perceived ease of use significantly predicted students’ attitudes toward IRS, which is consistent with TAM-based research showing that these two constructs are central to technology acceptance [9,46–52]. However, the present study also suggests that these constructs need to be interpreted in relation to the specific demands of EFL learning. In this context, perceived usefulness refers not only to whether a tool improves efficiency, but also to whether it supports participation, feedback, and understanding. Similarly, perceived ease of use refers not only to technical simplicity, but also to whether students can use the system without being distracted from language learning tasks.

Third, the non-significant role of prior technological experience contributes to a more situated understanding of technology acceptance in classroom-based EFL learning. The finding suggests that students’ attitudes toward IRS may depend less on their general digital familiarity and more on how the tool is pedagogically integrated into classroom practice. This has theoretical relevance because it shifts attention from technology readiness alone to the classroom conditions under which technology is experienced as useful, manageable, and supportive of learning. In other words, acceptance of IRS in EFL classrooms appears to be shaped by the interaction between technological perceptions and instructional design.

Finally, the study contributes to EFL technology research by connecting technology acceptance with classroom interaction. In language learning, students’ attitudes toward technology are closely related to how the tool affects participation pressure, feedback opportunities, confidence, and interaction with teachers and peers. The present findings therefore suggest that TAM-based explanations of educational technology use can be strengthened when they are interpreted alongside the affective and interactional realities of language classrooms. Rather than proposing a broad extension of TAM, the study offers contextual evidence for applying TAM to IRS-supported EFL instruction.

### Pedagogical implications

The findings have several pedagogical implications for EFL teaching and the instructional use of Interactive Response Systems. First, IRS should be integrated as part of lesson design rather than used as an isolated technological add-on. In this study, IRS activities were embedded into warm-up questions, comprehension checks, quizzes, live responses, feedback, and follow-up discussion. This suggests that teachers may benefit from using IRS to structure classroom participation and feedback throughout the lesson rather than limiting it to occasional testing or entertainment.

Moreover, IRS can be used to lower participation barriers in EFL classrooms. Because language learners may hesitate to respond publicly due to anxiety, limited confidence, or fear of making mistakes, response systems can provide a lower-pressure entry point for participation. Teachers can use anonymous or semi-anonymous responses, quick polls, and short comprehension checks to help students engage with classroom tasks before moving into oral discussion. This may be especially useful in EFL contexts where students are reluctant to speak in front of the whole class.

In addition, the findings suggest that teachers should pay attention to students’ perceptions of usefulness and ease of use when implementing IRS. The regression results showed that perceived usefulness and perceived ease of use significantly predicted students’ attitudes toward IRS. Therefore, teachers should make the purpose of each IRS activity clear, connect response tasks directly to learning objectives, and ensure that the system is simple enough for students to use without distracting them from language learning. If students perceive IRS activities as meaningful and manageable, they are more likely to develop positive attitudes toward the technology.

Furthermore, IRS-generated response patterns can be used as a formative feedback tool. Teachers can review students’ responses during class to identify misunderstandings, adjust explanations, and initiate peer discussion. This real-time feedback function may help teachers make classroom instruction more responsive to students’ needs. However, the effectiveness of this process depends on how teachers interpret and use the response data. IRS should therefore be accompanied by thoughtful questioning, clear feedback, and opportunities for students to discuss or revise their understanding.

Finally, institutions that intend to promote IRS-supported instruction should provide teacher training and technical support. Teachers need support not only in operating the platform, but also in designing effective questions, sequencing IRS activities, and using response data pedagogically. Without careful instructional planning, IRS may become a superficial tool for collecting answers rather than a meaningful support for participation, feedback, and interaction.

### Limitations

Several limitations should be acknowledged when interpreting the findings. First, the study used a quasi-experimental pre-test-post-test control-group design with one intact class representing each instructional condition. Because the IRS-supported condition and the traditional instruction condition each corresponded to a single class, class membership was fully confounded with instructional condition, and class-specific influences cannot be statistically separated from the observed condition differences. Although the two classes were broadly comparable in age, gender distribution, previous English performance, and pre-test attitude scores, these measured baseline similarities do not rule out unmeasured class-level differences. Accordingly, the findings should be interpreted as evidence of differential change between the two intact classes rather than as definitive evidence that the observed differences were caused solely by IRS-supported instruction. Future research should include multiple classes within each instructional condition and, where feasible, randomise at the class or individual level.

Second, the intervention lasted four weeks. Although significant improvements were observed across all eight attitudinal dimensions, the relatively short intervention period limits the extent to which the findings can be used to infer long-term effects. Students’ positive responses may partly reflect novelty effects associated with using a new classroom technology. Future studies should examine whether similar attitudinal improvements are sustained over a longer instructional period.

Third, the study relied on self-reported questionnaire data. Although the instruments demonstrated acceptable reliability and preliminary validity, self-report measures may be influenced by social desirability, response tendencies, or students’ awareness of participating in an intervention study. This may have introduced common method bias. Future research could combine questionnaire data with classroom observations, learning analytics, interview data, or behavioural indicators of participation to provide a more comprehensive account of IRS-supported learning.

Fourth, the regression analysis was conducted only with the IRS-supported group (n = 51), because students in the traditional instruction group did not use the IRS and therefore could not meaningfully evaluate its perceived usefulness or perceived ease of use. Although the regression model was statistically significant and the diagnostic statistics were acceptable, the relatively small regression sample limits the generalisability and statistical power of the predictor analysis. Future studies with larger samples should further examine the roles of perceived usefulness, perceived ease of use, and prior technological experience in shaping students’ attitudes toward IRS.

Finally, the study was conducted in one university EFL context in China. The findings may not be directly generalisable to other educational levels, institutional settings, language learning contexts, or IRS platforms. Future research should replicate the study across different universities, course types, learner populations, and technological platforms to examine whether the findings remain consistent across contexts.

### Suggestions for future research

Several directions for future research emerge from the present study. First, further work is needed across a wider range of educational contexts. The effectiveness of IRS is likely shaped by institutional conditions, classroom size, technological infrastructure, and local classroom culture. Future studies should examine its use in different universities, learner populations, and national settings. Such comparisons would clarify whether the present findings are context-specific or transferable across EFL environments.

Second, longitudinal research is needed to determine whether attitudinal benefits last over time. The present findings show clear short-term improvements in students’ attitudes. However, it is uncertain whether these effects persist. Future studies should investigate whether repeated and sustained IRS use leads to stable changes in participation, classroom interaction, and learner attitudes, or whether some effects diminish as the technology’s novelty fades.

Third, future research would benefit from mixed-methods designs to capture how and why the IRS influences learning. Quantitative approaches identify patterns and attitudinal shifts. Qualitative data, such as interviews, classroom observations, and reflective accounts, provide deeper insight into processes through which the IRS shapes participation, interaction, and affective experience. These methods help move beyond measuring IRS effectiveness, allowing research to explain its mechanisms.

Fourth, future studies should extend the theoretical framework by examining variables beyond the Technology Acceptance Model. While perceived usefulness and ease of use were significant, students’ responses to IRS are also shaped by factors like teacher facilitation, task design, learner autonomy, and classroom interactional patterns. Including these would help develop more comprehensive models for IRS use in EFL classrooms.

Finally, future research should examine how attitudinal outcomes relate to actual language-learning development. This study focused on students’ attitudes toward IRS. Further work should determine whether attitudinal improvements align with measurable gains in language proficiency, communicative competence, vocabulary, or speaking performance. Establishing these links would strengthen our understanding of IRS as not only a classroom tool, but also a pedagogical intervention with implications for language-learning outcomes.

## Conclusion

This study examined whether changes in university EFL students’ classroom attitudes differed between an IRS-supported intact class and a traditional-instruction intact class, and investigated whether perceived usefulness, perceived ease of use, and prior technological experience predicted students’ attitudes toward IRS. Using a quasi-experimental pre-test-post-test control-group design, the study compared an IRS-supported group with a traditional instruction group and further analysed TAM-related predictors within the IRS-supported group.

The findings showed that both groups reported more positive classroom attitudes after the instructional period. However, the IRS-supported group demonstrated significantly greater improvement across all eight attitudinal dimensions, including learning atmosphere, participation, attention, confidence, interactivity with teacher, interactivity with peers, learning support, and learning achievement/satisfaction. The significant Group × Time interaction effects indicated that the pattern of improvement differed between the two instructional conditions, with stronger gains observed in the IRS-supported group. Follow-up post-test comparisons further showed that students in the IRS-supported group reported more favourable attitudes than those in the traditional instruction group after the intervention.

The regression results showed that perceived usefulness and perceived ease of use significantly predicted students’ overall attitudes toward IRS, whereas prior technological experience did not. These findings suggest that students’ acceptance of IRS depended less on their general technological background and more on whether the system was experienced as pedagogically useful and easy to use in the classroom. In the EFL context, this means that IRS may be most positively received when it supports participation, feedback, comprehension checking, and classroom interaction without imposing unnecessary operational burden.

Overall, the study suggests that IRS-supported instruction may provide additional pedagogical support for improving university EFL students’ classroom attitudes when it is meaningfully integrated into lesson design. Rather than functioning merely as a response-collection tool, IRS can support participation, feedback, and interaction when used alongside clear instructional goals and teacher-guided discussion. However, given the quasi-experimental design, short intervention duration, and reliance on self-reported data, the findings should be interpreted cautiously as evidence of association rather than definitive causal proof. Because each instructional condition was represented by a single intact class, class-level influences cannot be separated from the instructional condition. Future research should examine IRS-supported EFL learning over longer periods, across different contexts, and with additional behavioural or qualitative evidence.

## Supporting information

S1 Data Raw data for the pre-test and post-test student attitude measures. This file contains the anonymised item-level and dimension-level data for the IRS-supported group and the traditional instruction group, including group membership, demographic and baseline information, and pre-test and post-test responses to the student attitude scale.
(XLSX)

S2 Data Raw data for the Technology Acceptance Model-related regression analysis. This file contains the anonymised data from the IRS-supported group used to examine perceived usefulness, perceived ease of use, prior technological experience, and overall attitude toward IRS.
(XLSX)

S1 File Completed PLOS questionnaire on inclusivity in global research. This file contains the completed PLOS questionnaire on inclusivity in global research.
(DOCX)
